# Atypical Renal Clearance of Nanoparticles Larger Than the Kidney Filtration Threshold

**DOI:** 10.3390/ijms222011182

**Published:** 2021-10-17

**Authors:** Christophorus F. Adhipandito, Siu-Hung Cheung, Yu-Han Lin, Si-Han Wu

**Affiliations:** 1 Graduate Institute of Nanomedicine and Medical Engineering, Taipei Medical University, Taipei 11031, Taiwan; christopherfidelone@gmail.com; 2 School of Medical Laboratory Science and Biotechnology, Taipei Medical University, Taipei 11031, Taiwan; m609108011@tmu.edu.tw; 3 Institute of Biomedical Sciences, Academia Sinica, Taipei 11529, Taiwan; dororo1910@gmail.com; 4 International Ph.D. Program in Biomedical Engineering, College of Biomedical Engineering, Taipei Medical University, Taipei 11031, Taiwan

**Keywords:** nanoformulations, inorganic nanoparticles, organic nanoparticles, renal clearance, glomerular filtration barrier, kidney filtration

## Abstract

In recent years, several publications reported that nanoparticles larger than the kidney filtration threshold were found intact in the urine after being injected into laboratory mice. This theoretically should not be possible, as it is widely known that the kidneys prevent molecules larger than 6–8 nm from escaping into the urine. This is interesting because it implies that some nanoparticles can overcome the size limit for renal clearance. What kinds of nanoparticles can “bypass” the glomerular filtration barrier and cross into the urine? What physical and chemical characteristics are essential for nanoparticles to have this ability? And what are the biomolecular and cellular mechanisms that are involved? This review attempts to answer those questions and summarize known reports of renal-clearable large nanoparticles.

## 1. Introduction

The term “nanoparticles” (NPs) refers to various types of particulate materials with one dimension of a size smaller than 100 nm [1,2]. They are increasingly popular for use as drug delivery systems to overcome classical problems faced by most drugs such as low solubility [3,4], low bioavailability [5,6], non-specificity [7,8], and/or toxicity [9,10]. Therefore, each year there is an increasing trend of NP-based therapies being approved by governmental medicine regulatory agencies such as the US Food and Drug Administration (FDA) [11,12] or the European Medicines Agency (EMA) [13,14]. However, even though many NP systems were designed to evade drug delivery-related problems, some types of NPs face unexpected challenges in vivo which can potentially lower their therapeutic effectiveness [15,16].

The term “nano-bio-interactions” is used to describe the various ways in which NPs interact with the body. These interactions can be categorized based on the organs which the NPs interact with, such as NP-liver interactions [17], NP-kidney interactions [18], NP-brain interactions [19], NP-tumor interactions [20], and other non-specific NP interactions [21]. When NPs are injected, the first interaction occurs when serum proteins circulating in the blood attach themselves onto the surface of the NP, forming what is known as a protein corona [22,23]. The characteristics of the corona formed will depend on the physical and chemical characteristics of the NP, such as its size, charge, and shape, which are discussed in more detail below.

The particles then circulate around the body in the bloodstream. Their biodistribution and other nano-bio-interactions are affected by their protein corona [24,25]. In some cases, the corona configuration can cause most of the NPs to be sequestered by the reticuloendothelial system (RES). This leads to their biodistribution mainly accumulating in the liver and spleen [26,27], which could lower their therapeutic effectiveness. To overcome this, scientists employ strategies such as conjugation of polyethylene glycol (PEG) to the NP surface. PEG helps reduce the formation of the protein corona, hence giving them a “stealth” effect to evade the RES and prolong their circulation time in vivo [22,26,28,29]. Another strategy to prevent the adsorption of proteins onto NP surfaces is to conjugate the surfaces of the particles with zwitterions [30].

After overcoming NP-liver interactions and those with other RES organs, subsequent obstacles are NP-kidney interactions [21]. It is well-known that, in general, NPs sized larger than 8 nm cannot be cleared out by the kidneys into the urine, as they are too large [31]. This is because large particles are generally unable to penetrate the glomerular filtration barrier (GFB) of the kidneys. The GFB functions as a sieve that normally only lets water and small solutes pass through [32,33]. Thus, it was assumed that “large” NPs could easily avoid clearance by the kidneys. Despite this assumption, there are reports of large NPs being found in the urine of lab mice [34,35,36,37]. There are even instances of particles being detected in cellular structures of the kidneys which are located beyond the GFB [35,38,39,40]. This is strange because normally NPs are degraded by the body [41,42].

Certain publications indicated that it is possible for some large NPs, even ones sized 100–200 nm [36,40], to escape degradation and then bypass the GFB. However, the cellular and molecular mechanisms of how these phenomena occur remain somewhat unknown. In this review, we attempt to gather known publications that show that “large” NPs can bypass the GFB. Large NPs, in this case, mean “NPs with a diameter of more than 8 nm”. Small particles with diameters of less than 8 nm are not considered because they easily cross the GFB. In addition, a summary of the physicochemical characteristics of large NPs is provided. This is done to examine which factors possibly impart the ability of large particles to be cleared into the urine.

## 2. The Glomerular Filtration Barrier

To investigate urinary excretion of NPs, understanding how urine forms is crucial. The process occurs in the kidneys [43,44] which are responsible for filtering the blood by transferring metabolic wastes from blood into the urine [45,46]. First, blood enters each kidney through a renal artery, which then branches off into much smaller blood vessels called renal arterioles. These arterioles eventually come into contact with nephrons, where filtration occurs [47]. The nephron is the primary “functional unit” of the kidney [48]. A single kidney of a human adult may contain 1–2 million nephrons [49].

**Figure 1:**
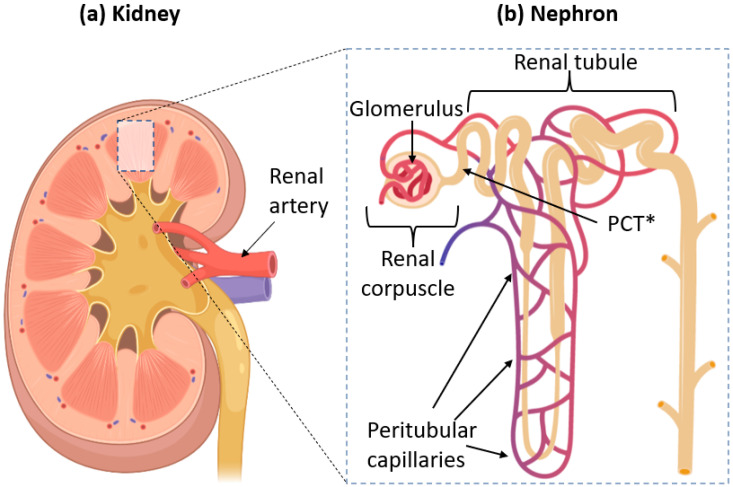
(**a**) Anatomy of a kidney, showing where one can find nephrons. Blood is supplied to the kidneys through the renal artery. (**b**) Structure of a single nephron. Filtration of blood occurs in the renal corpuscle. The renal artery splits into smaller vessels called arterioles, which form bundles of vessels inside the corpuscle called glomeruli. * PCT = Proximal Convoluted Tubule, the section of the renal tubule which is directly connected to the renal corpuscle. The whole of the tubules is surrounded by peritubular capillaries, which carry filtered blood from the glomeruli.

The nephron itself is divided into two main parts: the renal corpuscle, where the blood is filtered, and renal tubules, where essential molecules and ions are reabsorbed into the bloodstream. They are illustrated in Figure 1.

The renal corpuscle contains bundles of capillary vessels called glomeruli [50]. The name comes from Latin glomus meaning “ball of string”, entirely encapsulated by Bowman’s capsule. Blood flows from the renal artery into the glomeruli to be filtered. The filtrate is then collected by Bowman’s capsule, which directs it into renal tubules while the filtered blood exits the glomeruli. This anatomy allows the glomerulus and capsule to maximize the available surface area for efficient filtration [51]. The filtrate undergoes reabsorption in the renal tubules before finally exiting through the collecting duct to be eventually excreted out of the body as urine.

**Figure 2:**
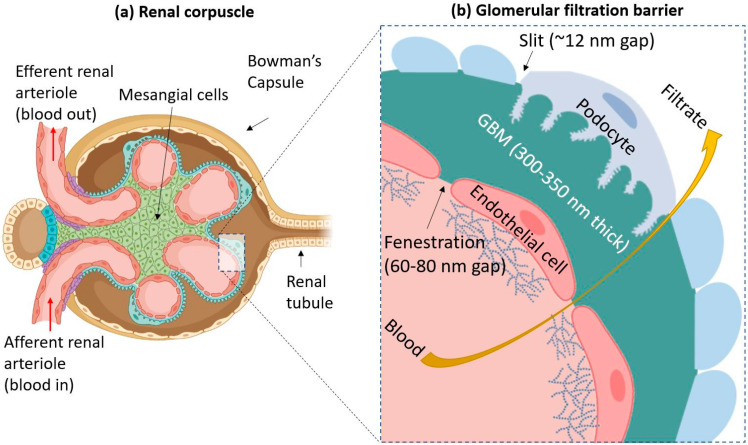
(**a**) A simplified diagram of the renal corpuscle. Blood flows into a glomerulus from the afferent renal arteriole. The filtered blood goes out through the efferent arteriole. Mesangial cells form the center of the corpuscle and hold the glomerulus together. (**b**) The three components of the glomerular filtration barrier (GFB) are endothelial cells (which have a surface covered by glycocalyx), the glomerular basement membrane (GBM), and podocytes. Blood from the glomerular capillary is filtered through these three layers, and the filtrate escapes out into the urinary space of Bowman’s capsule, as indicated by the yellow arrow. The GFB typically prevents particles larger than 6–8 nm in size from passing through.

The process of filtration can be further understood by examination at the cellular level. The mesangium and mesangial cells structurally support the center of glomeruli [52,53]. The sides of glomeruli which do not face the mesangium are covered in a layer of extracellular matrix (ECM) called the glomerular basement membrane (GBM). Directly above the GBM, there are specialized cells that engulf the glomerulus called podocytes. Therefore, the three layers involved in the filtration of blood are endothelial cells that form the capillaries of the glomerulus, the GBM, and podocytes. These three layers make up the glomerular filtration barrier (GFB), which determines which molecules can be filtered from the blood [54]. This is illustrated in Figure 2.

The GFB allows water and certain small to medium-sized solutes to freely pass through it, but larger molecules such as proteins and charged particles are generally prevented from passing [55]. This is due to the GFB’s unique architecture. First, endothelial cells of the glomerular capillaries possess a glycocalyx composed of glycoproteins. These filamentous structures on the cell surface help prevent the leakage of proteins, such as albumin, into the urine during normal conditions [56]. The glomerular endothelial cells also possess many pores in their cellular structure called fenestrations. Each fenestration is around 60–80 nm in diameter [57,58].

The second layer is the GBM, a specialized ECM between endothelial cells and podocytes. Its thickness is around 300–350 nm, and it is mainly composed of such proteins as laminin, nidogen, collagen IV, and agrin [59]. The GBM has a net negative charge due to the presence of agrin, and studies showed that positively charged and neutral molecules are more easily able to cross the GBM than negatively charged ones [60].

The final layer is composed of podocytes: octopus-shaped cells which wrap around the glomeruli [61]. The cells possess “foot processes”, which intercalate with other podocytes, forming “slits” [62]. These slits have porous diaphragms. The average radius of these pores is around 12 nm [63]. The podocytes also have a layer of glycocalyx above them.

Altogether, the three components of the GFB should prevent the leakage of molecules larger than 6–8 nm into the urine. However, recently, several publications showed the presence of NPs larger than the supposed size limit in urine [36,37].

## 3. Renal Clearance through Interactions with the GFB

Typically, particles with a diameter larger than 6–8 nm cannot be filtered out into the urine because the GFB repels them [31]. Despite this, there are cases where NPs can interact with the GFB. Zuckerman and Davis successfully synthesized 70-nm NPs made of small interfering RNA and cyclodextrin-containing polymer (CDP) NPs, which were able to cross the glomerular endothelium and be deposited in the GBM [64]. The particles did cross the podocyte layer as they remained within the GBM.

An example of NPs that can penetrate all three layers of the GBM is the oligoclusters synthesized by Lawrence et al. [34]. To summarize, they synthesized sodium borohydride (NaBH4) NPs with a molecular mass of 45 kDa and sodium thiocyanate (NaSCN) oligoclusters with molecular weights of 66 and 300 kDa. The oligocluster surfaces were coated with glutathione. After injecting them into mice, particles were detected in the urine. Through TEM imaging, they demonstrated that the ability of the NPs to penetrate the layers of the GFB depended on their size. The smaller-sized particles were distributed across all three layers. In contrast, the largest ones tended to accumulate in the endothelium (although a small amount did manage to cross into the podocyte layer as well) [34].

Recently, Fan and colleagues synthesized a nano-delivery system capable of targeting podocytes, where its cargo could then be released with the use of ultrasound (controlled-release) [65]. This nano-delivery system consisted of liposomes with a cargo of perfluoropentane (PFP) and dexamethasone. The surface of the liposomes was functionalized with PEG and BMS-α, a targeting ligand that selectively binds to the melanocortin-1 receptor (MC-1R). This receptor is expressed in high amounts by podocytes. TEM imaging showed that NPs had an average diameter of around 190 nm and could be endocytosed by podocytes in the glomeruli, indicating that they could penetrate the other two layers of the GFB [65]. It should be noted that nano-delivery systems had penetrated the podocyte layer in mouse models that had been induced with passive Heymann’s nephritis, which simulates leaky glomeruli of patients with membranous nephropathy. Thus, it is still unclear whether these NPs can penetrate the GFB in healthy glomeruli.

The glomerulus becomes leaky when the subject is experiencing diseases that lead to nephrotic syndrome. The leakiness causes proteinuria, which refers to the excretion of proteins (albumin) into the urine [66]. Nephrotic syndrome is the result of other diseases, such as an autoimmune disease (membranous nephropathy) [67], kidney damage due to high amounts of reactive oxygen species caused by high glucose levels (diabetic nephropathy) [68], and many more. It is much easier for NPs to be cleared into the urine due to the leaky glomeruli in subjects that have these glomerular diseases. However, except for the previously mentioned paper by Fan and colleagues (2021), most NPs mentioned in this paper can undergo renal clearance in healthy non-leaky kidneys.

Some types of NPs, like the 75 ± 25 nm PEGylated gold (Au-PEG) NPs synthesized by Choi et al., do not escape into the urine filtrate but are instead taken up by the mesangium within glomeruli. These NPs penetrate the first layer (the endothelium) then head to the mesangial cells. The Au-PEG NPs have been shown to be capable of diffusing into the mesangium [69]. This is possible because, unlike the three layers of the GFB, which lead into the urine, the barrier between the capillaries and the mesangium is only one layer of endothelial cells. Thus, some types of NPs of up to 80 nm in diameter can penetrate the mesangium [64].

Lastly, we must mention the unique way that single-walled carbon nanotubes (SWCNTs) interact with the GFB. SWCNTs are hollow tubes made up of a single layer of covalently-bonded carbon atoms. The length of an individual tube can be customized for the purposes of drug delivery (usually 100–500 nm), but their diameters remain small at around 1 nm [70]. Due to their thinness, they are able to rotate so that they can pass through the various pores and slits of the GFB, akin to how a needle would pass through a hole, and then eventually escape into the urine [71].

## 4. Bypassing the GFB through the Proximal Convoluted Tubules (PCTs)

One possible way that large NPs can bypass the GFB size limit is by going through the PCTs. The PCTs are the part of the renal tubule that is closest to the renal corpuscle. Its job is to reabsorb beneficial ions (e.g., sodium, chloride, and carbonate) from the glomerular filtrate [72]. These ions are returned to the bloodstream via peritubular capillaries that crisscross and form a network around the renal tubules [73]. As previously shown in Figure 1b, we can see the illustration of proximal convoluted tubules (PCTs) and peritubular capillaries in relation to the nephron.

The renal tubule’s job is to reabsorb beneficial ions from the filtrate. The PCTs are the part of the tubule that is immediately next to the renal corpuscle. The tubules are surrounded by peritubular capillaries which carry filtered blood from the glomeruli. Large nanoparticles that cannot cross the GFB end up flowing into the peritubular capillaries, and when they come in contact with PCT cells, they can get transcytosed into the urinary space.

Naumenko et al. demonstrated an example of this transcytosis by synthesizing 140-nm iron oxide nanocubes and nanoclusters. They were injected into mice, and a few hours later, they were detected intact within the urine (shown by transmission electron microscopy (TEM)) [35]. The nanocubes and clusters were also conjugated with fluorescent Cy5 dye for detection using intravital microscopy (IVM). During live in vivo experiments, fluorescence was shown in the PCTs instead of the glomeruli. This suggests that iron oxide NPs experience transcytosis in the PCT region.

In addition, experiments carried out by Williams et al. also led them to conclude that their NPs underwent transcytosis across the peritubular capillaries. Briefly, they synthesized poly(lactic-co-glycolic acid) (PLGA) particles conjugated with polyethylene glycol (PEG) (PLGA-PEG) that had a diameter of 350–400 nm. Although these NPs were not found intact in the urine of the mice, IVM experiments revealed that they too had accumulated in PCT cells [40].

Previous studies showed that certain virus particles, such as cytomegaloviruses, can be renally cleared through the urine even though they are large (100–240 nm) [74]. Wyss and colleagues (2019) believed that glycoproteins found on the virus surface help them escape into the urine intact. Therefore, they synthesized PLGA-based NPs, which were functionalized with glycosaminoglycan on the surface to mimic the surface of viruses. The resulting particles had diameters of around 130–180 nm. Through fluorescence molecular tomography, they showed that these particles had accumulated in the bladder of a mouse a few hours after injection. They also suspected that the mechanism was due to NPs crossing from peritubular capillaries into renal tubules [75]. Unfortunately, they did not provide clear evidence, such as TEM imaging of particles in the urine, to show that their NPs had undergone renal clearance. We do not know if the particles had degraded or if they remained intact.

To conclude this section, we must refer to the single-walled carbon nanotubes (SWCNTs) that were mentioned previously. Alidori and colleagues (2016) had synthesized special SWCNTs that were functionalized with ammonium to deliver small interfering RNA (siRNA) into PCT cells, which they call fCNT/siRNA. Staining and imaging experiments confirm that while a majority of the nanotubes had been eliminated into the urine, some of them had been uptaken by the PCT. Experimental data suggest that uptake was mediated through a clathrin-mediated pathway [76].

## 5. Renal Clearance through an Unknown Route

Some types of silica NPs were shown to be able to undergo renal clearance without being degraded, but their route of exit remains undiscovered [77,78,79]. A recent example is mesoporous silica NPs (MSNs) synthesized by Dogra and colleagues [36]. They synthesized particles with several diameters (32–162 nm), whose surfaces were coated with different types of functional groups such as trimethylsilane (TMS), polyethyleneimine (PEI), or quaternary ammonium (QA) to see the effects of size and charge on the particle biodistribution after they had been injected into mice. By using single-photon emission computed tomography (SPECT)/CT, they showed that certain types of MSNs could accumulate in the bladder. Furthermore, a TEM analysis of the urine showed intact NPs [36]. However, the authors noted that further research was needed to elucidate how these large MSNs could cross into the urine.

Similarly, Pérez-Campaña et al. synthesized radioactive nitrogen-labeled aluminum oxide (13N-labelled Al_2_O_3_) NPs, which could be tracked in vivo using positron emission tomography (PET) [37]. Biodistribution studies showed that some particles accumulated in the kidneys and bladder: NPs had diameters of 10, 40, and 150 nm. As expected, the amount of NPs detected in those organs decreased as the particle size increased [37]. The authors claimed that the 10-nm particles could pass through the glomerulus to reach the urine, but they did not carry out any studies to confirm this. In addition, they did not explain how the larger 40- and 150-nm particles could escape into the urine as well, meaning that the renal clearance mechanism for these types of NPs is still unknown.

## 6. Possible Mechanisms of Renal Clearance

As previously mentioned, most large NPs (which have diameters of >8 nm) can escape into the urine via two main routes, namely by interacting with the GFB or bypassing the GFB and going through the PCT. This section explains in more detail the possible molecular and cellular mechanisms by which the particles can be renally cleared.

We mentioned that the GFB’s function was to prevent leakage of proteins into the urine. Despite this, albumin, the most common protein found in the blood [80], can cross the GFB when logically, it should not be able to. For humans, every day, about 3.3 g of albumin passes through the GFB, and almost all of it is reabsorbed back into the bloodstream through the renal tubules [81].

More specifically, a lot of albumin reabsorption occurs in the PCT. There are two known mechanisms for this process: (1) albumin binding to the megalin/cubulin receptor complex found on PCT cell surfaces, which captures the proteins via a clathrin-mediated pathway, and (2) a non-selective process that takes in albumin (as well as other molecules) in large amounts, described as “fluid-phase endocytosis” which has not yet been extensively characterized [82]. In addition, the FcRn receptor plays a crucial role in ensuring that endocytosed albumin is not degraded but is instead safely transported out of the cell [83].

On the other hand, it may be possible that large NPs within the renal capillaries can take the opposite route, which is to say that they get transcytosed from the renal capillaries, pass through the PCT, and into the urine filtrate. But as far as we know, there are no studies that prove this. Nonetheless, authors like Williams and colleagues hypothesized that their NPs can accumulate in PCT cells through some sort of transcytosis process [40]. Naumenko et al. note that transcytosis of their NPs into the PCT may be explained by a hypothesis from the previously mentioned paper by Williams and colleagues. They hypothesize that NPs can be transcytosed into the PCT due to the sharp drop in blood pressure in the nephron and the considerable absorptive pressure of peritubular capillaries [35,36,37,38,39].

The role of pressure in the peritubular capillaries towards the transport of NPs is still unclear. Pallone and Cao (2008) list various papers which show that changes in the oncotic pressure of the peritubular capillaries may affect the reabsorption of fluids in the PCT [84]. Briefly, they postulate that the addition of hyperoncotic fluid in the peritubular capillaries may affect the reabsorption of fluids or solutes by the PCT. When the NPs are located in the peritubular capillaries, they increase the oncotic fluid, explaining their transport into the PCT.

Besides the PCT, some types of NPs can go through the GFB by passing through podocytes. Lawrence and colleagues showed this with TEM images of their NPs in the process of being transported through the three layers of the GFB. They concluded that NP size affects their penetrative ability, with smaller NPs being able to penetrate more easily. Furthermore, they conducted experiments using fluorescent-tagged albumin of different sizes. Those experiments give similar results, meaning that the penetration of albumin was size-dependent [34]. This may mean that the molecular mechanism of NP transcytosis across the podocyte barrier (the GFB) may be similar to the one taken by albumin.

**Figure 3:**
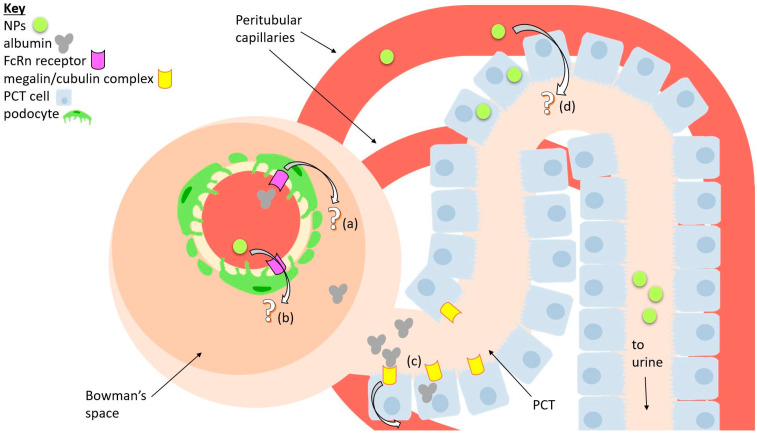
A diagram of a simplified nephron that summarizes possible mechanisms of transport for nanoparticles (NPs) and albumin. (**a**) A possible way albumin may be transcytosed from glomerular capillaries into tubules is through the FcRn receptor, which is expressed by podocytes. (**b**) Similarly, NPs may perhaps take the same route. (**c**) Reabsorption of albumin back into the bloodstream through interactions with the megalin/cubulin receptor complex, expressed by proximal convoluted tubule (PCT) cells. (**d**) A possible pathway for NPs being transcytosed via PCT cells is possibly also due to large absorptive pressure.

Unfortunately, the precise molecular mechanism by which albumin is transcytosed through the podocytes is not yet fully understood. Dobrinskikh et al. conducted in vitro tests using human urine-derived podocyte-like epithelial cells (HUPECs) and concluded that albumin is transported via interactions with the FcRn receptor and caveola-dependent endocytosis [85]. However, other studies suggested that different pathways may be involved, so the exact mechanism is not fully understood. What can be theorized is that the transcytosis of albumin through podocytes and its subsequent reabsorption in the PCT may be a natural system which prevents the “clogging” of the GFB by proteins [86]. Thus, the phenomenon of large NPs being able to overcome the GFB is perhaps due to NPs being able to access this system. The possible mechanisms of transport are summarized in Figure 3.

## 7. NP Physicochemical Properties Which Allow for Renal Excretion

A summary of several large NPs that can cross the GFB, bypass the GFB, and/or escape into the urine is provided in Table 1. These particles all have different characteristics. In this section, we attempt to identify the physical and chemical characteristics of NPs underlying their differences in distribution and clearance.

## 8. The Effect of Nanoparticle Size on Renal Excretion

The exact relationship between the ability to overcome the GFB and the size of the NP is still unclear. Larger particles seem to have a harder time directly penetrating the layers of the GFB, as shown by the experiments performed by Lawrence et al. (2017) [34]. On the other hand, there are some examples of NPs over 100 nm in size which cannot cross the GFB, but bypass it through the PCT and then escape into the urine [35,39,40]. Furthermore, experiments by Dogra and colleagues (2018) using mesoporous silica nanoparticles did not show any correlation between NP size and their accumulation in the bladder [36].

The size of the nanoparticle may affect processes relating to endocytosis by the renal cells. Hoshyar et al. (2016) reviewed publications which studied the in vitro cellular uptake of several types of NPs and of different sizes. They concluded that a diameter of around 30–60 nm for spherical NPs is the optimal size to encourage the process of cell membrane-wrapping [87]. Particles which have a diameter less than 30 nm are too small to effectively activate the membrane-wrapping process, while NPs larger than 60 nm in diameter are prone to steric hindrance as well as receptor saturation. Unfortunately, none of the studies had used neither podocytes nor PCT cells, and they were all conducted in vivo, so it is unknown if this size rule would apply for in vivo renal clearance.

A review by Pearson and colleagues (2014) summarized that NPs measuring about 60 nm tend to experience caveolae-mediated endocytosis, while those measuring about 120 nm undergo clathrin-mediated endocytosis [88]. Caveolae-mediated endocytosis may be important for transcytosis, as Moriyama and colleagues (2018) note that caveolae-mediated transcytosis (with the help of the FcRn receptor) plays a critical role in the transcytosis of albumin [89].

## 9. The Effect of Nanoparticle Charge on Renal Excretion

The electrical charge of NPs can affect its clearance in two ways: (1) first, serum proteins may interact with the charged NP to form a protein corona and hence increase the particle’s hydrodynamic diameter (NP size), and (2) the charged particles may interact with the charges found in the cellular structure of the kidney [90]. Most publications usually state that positively-charged and neutrally-charged NPs will find it easier to cross the GFB. This is because negatively-charged particles will experience a repulsive force from the negative charges of the glycocalyx of epithelial cells and of the proteins in the GBM [21,60].

To demonstrate this, Dogra et al. (2018) compared the distribution of 50 nm MSNs functionalized with either TMS (neutral charge), QA (positive) or PEI (positive). Within 30 min after injection into the mouse, all of the particles had been detected in the bladder, showing no problem in overcoming the GFB [36]. Lawrence et al. (2017) showed that their (negatively-charged) NaBH_4_ particles accumulate near podocyte foot processes, but they are displaced by heparin when it is injected into the mice, due to heparin having a more negative charge [34]. Similarly, Liu and colleagues (2020) show that their negatively-charged polystyrene NPs (20–100 nm in size) tend to accumulate in the glomerulus [38]. Thus, it would appear that positively-charged and neutral NPs are more favorable to experience renal clearance, while the negatively-charged ones linger within the nephron structures. As a final note, Manzanares and Ceña (2020) observed that positive NPs tend to be easier to be endocytosed due to the cells having a negative surface charge [91].

## 10. The Effect of Nanoparticle Composition on Renal Excretion

Du and colleagues (2018) suggest that NPs of the same size but composed of different materials (in other words, different densities) will have different clearance properties. For metallic NPs, the denser materials will be harder to be cleared out. They also suggest that the clearance of organic NPs will be easier [21]. However, it is also important to note that their conclusions apply to small (<8 nm) particles, while this review mostly focuses on the clearance of large NPs with diameters larger than 8 nm.

As we have seen in Table 1, large inorganic NPs (made of silica, iron oxide, sodium borohydride, etc.) as well as organic NPs (polymer-based NPs, liposomes) have been reported to be able to cross the GFB to varying degrees. One interesting note is that the organic-based NPs are mostly designed to be taken up by the renal cells and accumulate in the glomerulus. We can assume that inorganic-based materials are more suitable for designing renal-clearable NPs.

## 11. The Effect of Nanoparticle Surface Modifications on Renal Excretion

As mentioned earlier, the most common surface modification for NPs is PEGylation, which serves to protect them from the reticuloendothelial system and prolong their circulation time. The NPs listed in Table 1 show that some large NPs, which are not yet PEGylated, still have the ability to bypass the GFB and some can be cleared out into the urine. Thus, it is not clear whether PEG functionalization contributes to the renal clearance of large NPs.

On the other hand, the surface modifications for the NPs may include the addition of charged functional groups which give the NPs a surface charge. This is directly related to the previous point about nanoparticle charge.

**Figure 4:**
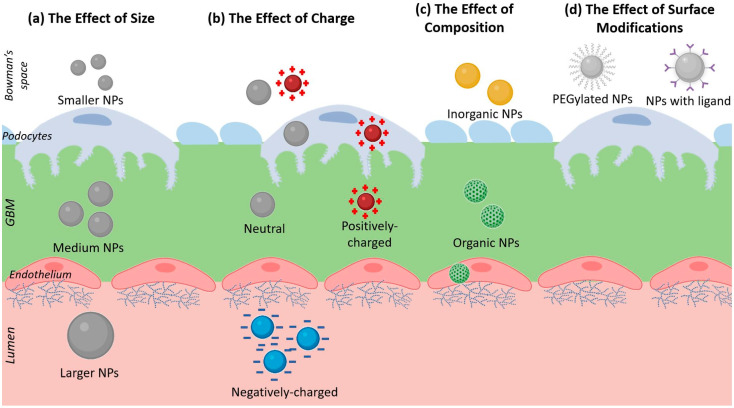
A diagram showing the 4 main factors which may give NPs the ability to bypass the GFB: (**a**) Size, showing NPs of different diameters. (**b**) Charge, showing NPs with different electrical charges. (**c**) Composition, showing organic and inorganic NPs. (**d**) Surface modifications, showing PEGylated NPs and NPs with ligand functionalization.

Lastly, the functionalization of targeting ligands on the surface of NPs will enable the uptake of NPs by certain cells. For example, podocytes express FcRn receptors which play a role in the uptake of macromolecules such as albumin. Wu and colleagues (2017) synthesized NPs by conjugating bovine serum albumin with methylprednisolone (BSA-MP) [92]. Biodistribution studies show that most of their particles accumulate in the liver and in the kidneys 24 h after injection in mice. Besides that, in vitro experiments with human podocyte cells and HK-2 show that the BSA-MP particles are uptaken by them. Theoretically, it may be possible that NPs functionalized with other targeting ligands can aid in the transcytosis of NPs into the urinary space, although so far there are no publications to conclusively prove this. Figure 4 summarizes the four main physico-chemical characteristics which may contribute to NP renal excretion:

## 12. Conclusions

The ability of certain nanoparticles which have diameters of over 8 nm to bypass the glomerular filtration barrier is a very unique phenomenon. Some of them are able to accumulate in different parts of the glomerulus, while others are even able to escape into the urine. The abilities of these NPs to either fully experience renal clearance or accumulate in certain parts of the kidney is dependent on their various physico-chemical characteristics. To the best of our knowledge, this is the only review which has attempted to compile publications that report renal-clearable NPs and categorize them.

Further studies are required in order to get a more complete understanding on how the physico-chemical characteristics are able to affect the NPs. This is necessary as it would be useful in designing specialized NPs for treating certain diseases. For example, particles could be engineered to specifically deliver the drugs to the podocytes or mesangium. Alternatively, other NPs can be designed specifically to avoid accumulation in the GBM in order to prevent renal toxicity.

It is important to note that these physico-chemical characteristics are by no means exclusive. For instance, the addition of targeting ligands as surface modifications may also alter the surface charge and diameter of the particle. Thus, it is difficult to pinpoint exactly which characteristics are solely responsible for what. The ability of NPs to bypass the GFB is a result of a combination of various characteristics.

Lastly, the exact cellular and molecular mechanisms of how some of these particles are able to either accumulate in the glomerulus or escape into the urine are still not fully understood. Therefore, it is important to conduct further research with other types of NPs in order to understand how NPs and the kidneys interact.

## Figures and Tables

**Table 1:** A summary of known large NPs which have the ability to either accumulate in certain parts of the kidney or fully experience renal clearance.

NP System	Size(s)	Renal Clearance Route	Reference
Small interfering RNA and cyclodextrin-containing polymer (siRNA/CDP) NPs	70 nm	Directly cross the GFB, but accumulate in the GBM	Zuckerman and Davis (2013) [64]
Sodium borohydride (NaBH_4_) NPs with GSH surface modification	45 kDa	Directly cross the GFB	Lawrence et al. (2017) [34]
Sodium thiocyanate (NaSCN) oligoclusters with GSH surface modification	66 and 300 kDa	Directly cross the GFB	Lawrence et al. (2017) [34]
Single-walled carbon nanotubes functionalized with ammonium and siRNA (fCNT/siRNA)	Length 300 nm, diameter around 1 nm	Directly cross the GFB, most of them cleared into urine but some reabsorbed by PCT	Alidori et al. (2016) [76]
Nano-delivery system consists of liposome containing PFP and Dex. Surface functionalized with PEG and BMS-α	190 nm	(Presumably) directly cross GFB, taken up by podocytes	Fan et al. (2021) [65]
PEGylated gold nanoparticles (Au-PEG NPs)	75 ± 25 nm	Cross endothelium and accumulate in mesangium	Choi et al. (2011) [69]
Polystyrene PEG-carboxylate NPs	20 and 100 nm	Accumulate in glomerulus, some in renal tubules	Liu et al. (2020) [38]
Iron oxide nanocubes and nanoclusters	140 nm	Through PCT	Naumenko et al. (2019) [35]
Poly(lactic-co-glycolic acid) particles conjugated with PEG (PLGA-PEG)	350–400 nm	Through PCT	Williams et al. (2018) [40]
PLGA-based and they were functionalized with glycosaminoglycan	130–180 nm	(Presumably) through PCT	Wyss et al. (2019) [75]
MSN-PEG functionalized with TMS, PEI or QA	32–162 nm	Unknown	Dogra et al. (2018) [36]
Radioactively-labelled aluminum oxide nanoparticles (13N-labelled Al_2_O_3_ NPs)	10 nm, 40 nm and 150 nm	Unknown	Pérez-Campaña et al. (2013) [37]
